# K_ATP_ Channel Function: More than Meets the Eye

**DOI:** 10.1093/function/zqab070

**Published:** 2022-01-10

**Authors:** Show-Ling Shyng

**Affiliations:** Department of Chemical Physiology and Biochemistry, School of Medicine, Oregon Health and Science University, Portland OR 97225, USA

ATP-sensitive potassium (K_ATP_) channels are ATP- and ADP-regulated potassium channels that control membrane potential-dependent physiological processes to meet metabolic demands. The first K_ATP_ channel was identified in cardiac muscle in 1983 as an outward K^+^ current induced by cyanide or hypoxia, which was highly sensitive to inhibition by intracellular ATP. Related K_ATP_ channels have since been found in many other tissues and cells including pancreatic endocrine cells, skeletal muscle, smooth muscle, neurons, glial cells, and kidney. At the molecular level, all K_ATP_ channels are composed of two gene products, the pore-forming K^+^ channel subunit Kir6, and the sulfonylurea receptor SUR, an obligate regulatory subunit. In humans, these are expressed from two alternative pairs of genes. The first pair, *ABCC8* and *KCNJ11* on chromosome 11 encode SUR1 and Kir6.2, respectively. The second pair, *ABCC9* and *KCNJ8* on chromosome 12, encode SUR2 and Kir6.1, respectively; SUR2 transcripts are alternatively spliced to form variants SUR2A and SUR2B. Each K_ATP_ channel is a hetero-octamer containing four pore Kir6.x and four SURx proteins. In recombinant expression systems, assembly of various Kir6.x and SURx generates different flavors of K_ATP_ channels with distinct nucleotide sensitivities and pharmacology, accounting for much of the phenotypic variation between native channels in different tissues.

Our understanding of the physiological roles of K_ATP_ channels is a work in progress that has evolved with cloning of the channel genes, molecular and biochemical analyses, transgenic animal studies, and discovery of genetic mutations in K_ATP_ channel genes in human diseases. In key early work, congenital forms of hyperinsulinemia and hypoglycemia were linked to loss of function mutations in *KCNJ11* and *ABCC8*, encoding the pore Kir6.2 and regulatory SUR1 subunits of the pancreatic β-cell K_ATP_ channel, which indicated this channel plays a critical role in regulation of insulin secretion.^1^ Subsequently, gain-of-function mutations in the same genes were shown to underlie a spectrum of neonatal diabetes,^2,3^ which solidified the essential role of K_ATP_ channels in glucose-stimulated insulin secretion. In addition to neonatal diabetes, some mutations in the pancreatic K_ATP_ channel were further associated with developmental delay, epilepsy, skeletal muscle dysfunction, referred to as DEND or iDEND (intermediate DEND) syndromes, differing in severity.^4^ Transgenic expression studies to determine whether defects beyond the pancreas stem from overactive channels within other tissues, or rather reflect systemic glucose dysregulation have proven insightful. In 2010, it was reported that transgenic mice expressing a human iDEND Kir6.2 mutation (V59M) recapitulated characteristic muscle dysfunction when transgene expression was targeted to neuronal cells rather than skeletal muscle.^5^ As the mutant transgene was not expressed in pancreas, the study suggests independent physiological functions for Kir6.2-containing channels in the brain. Yet, the cognitive deficits observed in some patients with *KCNJ11* or *ABCC8* mutations have remained unexplained, until recently. By introducing into mice a mutated Kir6.2 known to cause neonatal diabetes in mice, but driven by a tissue-specific inducible promoter, Yahil et al. showed in a battery of behavioral tests that while pan-neuronal expression of the mutant led to sensorimotor as well as cognitive deficits, expression of the mutant transgene expression specifically in hippocampal neurons resulted in impaired learning and memory, with reduced long-term potentiation observed in hippocampal neurons.^6^ Importantly, mice in which expression of the mutant channel was restricted to pancreatic β-cells only exhibited diabetes without any neuronal phenotype. This work has provided the most definitive evidence to date that cognitive deficiency in DEND results directly from overactive neuronal K_ATP_ channels in brain neurons.

Clarifying the physiological functions of K_ATP_ channels in the cardiovascular system has remained an active focus of research since their initial discovery in cardiac myocytes. The predominant isoform composed of Kir6.2 and SUR2A appears little involved in heart function under normal physiological conditions but important for sustaining cardiac function under extreme metabolic stress, such as ischemia, hypoxia, and prolonged exercise. Although additional K_ATP_ isoforms are expressed in the heart, their precise composition, expression patterns, and functions are not resolved. In humans, variants in all four K_ATP_ genes have been reported in a number of cardiovascular diseases. While a contribution of K_ATP_ channel dysfunction to the observed pathologies is not well established in most cases, a convincing causal association is seen between gain of function mutations in *ABCC9* and *KCNJ8* and Cantú syndrome, a pleiotropic developmental disorder including severe cardiovascular defects, hypertrichosis, facial dysmorphy, and macrocephaly. Correlation of genetics and clinical phenotypes in Cantú patients, together with studies of transgenic mice expressing Kir6.1 and SUR2 with human Cantú mutations provide compelling evidence that those mutations in Kir6.1 and SUR2B that produce overactive vascular smooth muscle K_ATP_ channels cause systemic hypotension, which leads to the wide range of pathologies presenting in Cantú syndrome. Very recently, York et al. reported that mice carrying Cantú K_ATP_ mutations also exhibit reduced intestinal contractility, potentially accounting for the gastrointestinal dysmotility frequently seen in Cantú patients.^7^ This study uncovers a previously unappreciated role of the Kir6.1/SUR2B channel in the gastrointestinal tract, raising the possibility that this channel holds additional functions yet to be discovered in smooth muscles in other tissues.

While the K_ATP_ channel physiology field has steadily matured in the past few decades, it is clear much more remains undeveloped. Several challenges have long impeded progress. First, the complexity of K_ATP_ compositions and expression patterns makes it difficult to ascribe physiological functions to functional properties of the channel subtypes. In some cases, co-assembly of multiple SURx and Kir6.x subunits may occur. Second, many studies in the literature have relied heavily on pharmacological agents for interpretation, despite lack of K_ATP_ channel isoform specificity for activators and inhibitors employed, which may also have off-K_ATP_ target effects. Third, interpretations of the relationship between genetic variants and the risk for certain diseases, or their pathological progression, are often freighted by modest functional deviations of the variant channels, which are compounded by limited sample sizes. For example, many studies have implicated the Kir6.2 E23K variant as a risk factor for type 2 diabetes. However, definitive mechanistic linkage has been difficult to establish, perhaps due to the relatively subtle effect of the variant on β-cell K_ATP_ channel activity. In a recent study of mice expressing the Kir6.2 E23K variant in β-cells, impairment in glycemic control was not apparent, unless challenged with a high fat diet and only in homozygous mutant animals.^8^

The horizon offers new tools and approaches to overcome these challenges. Advances in CRISPR technology has accelerated the development of genetic manipulations targeted to specific populations of cells, and will greatly improve our precision in delineating the physiological and pathological roles of K_ATP_ channels. Likewise the recent revolution in single-particle cryo-EM technique has begun to dramatically transform understanding regarding the molecular basis of channel function, regulation, and pharmacology. Already the structures of several K_ATP_ channels have been determined, variously bound with physiological and pharmacological ligands.^9^ These structures reveal a magnificent molecular pinwheel superstructure with four SUR subunits surrounding a Kir6 tetrameric pore, and the principal allosteric binding sites for nucleotides, drugs, and phospholipids. Moreover, conformational differences between different K_ATP_ channel isoforms and ligand-bound states as well as interactions between subdomains observed in molecular dynamics simulations provide tremendous mechanistic insights into channel function and regulation.^10^ These structures should form a new basis for the development of isoform-specific drugs or antibodies and nanobodies not only for probing channel structure and function in native tissues but also for treating disease caused by dysfunction of various K_ATP_ channels. Cross fertilization between different disciplines including human genetics, transgenic animals, biochemistry and biophysics will be key to illuminating the path forward. There is evidence that K_ATP_ channels are involved in immune response, glial cell function, skeletal muscle metabolism, kidney function, and more. Looking ahead, there is great optimism that exciting discoveries on new K_ATP_ channel functions will continue to emerge.
